# "Don't touch" bone lesions: how can we contribute?

**DOI:** 10.1590/0100-3984.2019.52.1e2

**Published:** 2019

**Authors:** Francisco Abaeté das Chagas-Neto

**Affiliations:** 1 PhD, Professor of Radiology at the Centro Universitário Christus, Fortaleza, CE, Brazil. Email: fabaeteneto@gmail.com. https://orcid.org/0000-0002-6987-2072.

The so-called "don't touch" bone lesions are typically identified as incidental findings on imaging exams. Most of these lesions are pseudotumors, benign bone lesions or anatomical variants.

As the name suggests, "don't touch" lesions do not require the use of biopsy or other invasive procedures. However, they can be misdiagnosed as aggressive neoplastic lesions, generating concern and leading to unnecessary procedures. Correct identification of these lesions, with a specific diagnosis, minimizes the morbidity and costs associated with their management^(^1^)^.

In most cases, the method used for the initial identification, characterization, and classification of "don't touch" lesions is conventional X-ray. The integrated analysis between the imaging findings and the clinical and laboratory data is essential^(^2^)^.

In some challenging situations, including uncommon presentations such as a hemophilic pseudotumor, computed tomography or magnetic resonance imaging can be used in order to characterize the lesion, thus increasing the reliability and confidence in the accuracy of the diagnosis^(^3^-^6^)^.

There is currently a tendency to use computerized systems to support the clinical decision and diagnosis, contributing to the management of medical knowledge. In a recent article addressing this theme, Moreira et al.^(^7^)^ described a cognitive mapping system specifically focused on supporting the diagnosis of solitary bone tumors in pediatric patients, with the objective of supporting the decisions of medical professionals and promoting training in the area, potentially reducing errors in the diagnosis of such lesions. Costa^(^8^)^ also recently discussed the application of artificial intelligence in the evaluation of bone tumors, highlighting the great potential of such tools. However, the author emphasized the importance of face-to-face physician-patient interaction in the overall approach to cancer patients.

In an article published in this issue of **Radiologia Brasileira**, Fonseca et al.^(^9^)^ have reviewed, in an illustrative way, the main bone pseudotumors and "don't touch" lesions. The authors addressed a variety of such lesions/conditions-cortical desmoids, subchondral cysts, costochondritis, small bone islands, fibrous dysplasia, non-ossifying fibromas, simple bone cysts, aneurysmal bone cysts, bone infarction, synovial cysts, melorheostosis, vertebral hemangiomas, discogenic vertebral sclerosis, myositis ossificans, and humeral pseudocysts-, highlighting their main characteristics on the various imaging methods.

Radiologists and radiology residents need to be familiar with the "don't touch" lesions and their different presentations in the various imaging methods. Thus, they can actively and efficiently contribute to the diagnosis and follow-up of these patients, avoiding unnecessary invasive procedures, reducing patient morbidity, and optimizing the use of health care resources.
